# Immunosuppression and Outcomes in Patients with Cutaneous Squamous Cell Carcinoma of the Head and Neck

**DOI:** 10.3390/clinpract15010021

**Published:** 2025-01-17

**Authors:** Doriana Iancu, Ana Fulga, Doina Vesa, Iuliu Fulga, Dana Tutunaru, Andrei Zenovia, Alin Ionut Piraianu, Elena Stamate, Corina Sterian, Florentin Dimofte, Mihail Alexandru Badea, Alin Laurentiu Tatu

**Affiliations:** 1 ENT Department, “Sfantul Andrei” Emergency Hospital of Galati, 800578 Galati, Romania; 2 Faculty of Medicine and Pharmacy, Dunarea de Jos University of Galati, 800010 Galati, Romania; 3 Department of Forensic Medicine, “Sfantul Andrei” Emergency Clinical Hospital of Galati, 800223 Galati, Romania; 4 Department of Otorhinolaryngology, “Cai Ferate” General Hospital, 800223 Galati, Romania; 5 Cardiology Department, Emergency University Hospital of Bucharest, 050098 Bucharest, Romania; 6 Danube Medical Center, 810197 Braila, Romania; 7 Department of Microbiology, George Emil Palade University of Medicine, Pharmacy, 540139 Targu Mures, Romania; 8 Department of Dermatology, “Sfanta Cuvioasa Parascheva” Hospital of Infectious Diseases, 800179 Galati, Romania; 9 Multidisciplinary Integrative Center for Dermatologic Interface Research, 800179 Galati, Romania

**Keywords:** immunosuppression, head and neck skin cancer, cutaneous squamous cell carcinoma

## Abstract

Cutaneous squamous scell carcinoma (cSCC) is a frequent non-melanoma skin cancer that originates from keratinocytes with increased prevalence. cSCC can be either in situ, as in Bowen’s disease, or extended. Advanced age, accumulated sun exposure, light pigmentation, and prior skin cancer diagnosis are all significant risk factors for cSCC. Although most cSCCs can be treated surgically, some recur and metastasize, resulting in death. The role of immune status is not yet determined in the prognosis of these patients. **Objective**. Immunosuppressed patients are more likely to develop cSCC, which is often characterized by more aggressive, multifocal lesions. This study aimed to determine the risks of mortality in patients with cSCC and immunosuppression versus non immunosuppression and to compare variations in overall survival based on different clinical features. **Method**. We evaluated clinical cases of patients at “Sfantul Apostol Andrei” Emergency Hospital of Galati, Romania, from 1 March 2018 to 1 April 2024. Subjects in the trial had to be at least 18 years old and have a pathologically confirmed diagnosis of cutaneous head and neck squamous cell carcinoma (cHNSCC). We divided the patients into two different categories based on whether they had immunosuppression. **Results.** In this cohort of 68 subjects with cSCC, patients with immunosuppression had significantly lower overall survival, as well as lower three- and five-year survival rates compared with those without immunosuppression, even after adjustment for age, sex, stage, and previous surgical treatment. The median survival time for immunosuppressed individuals ranged from 11 to 21 months, varying based on their particular characteristics, and most critically, on the presence of other malignancies, while that of immunocompetent patients ranged from 18 to 51 months. In addition, immune-deficient patients with early-stage disease had a 21-month median survival rate that changed to11 months for advanced-stage cases. In a similar manner, immunocompetent patients with early-stage cancer had a significantly better median survival than those withadvancedstages,43 versus 18months. Our results indicate that immunosuppression is a distinct risk factors associated with a less favorable outcome in patients with cHNSCC.

## 1. Introduction

Non-melanoma skin neoplasms commonly include basal-cell and squamous cell carcinomas. Squamous histology is associated with a less favorable outcome than that of basal-cell carcinoma (BCC), with a higher risk of regional recurrence and, sometimes, nodal and metastatic dissemination [1]. Persistent interaction with ultraviolet light can cause chronic cutaneous damage and cancer in susceptible areas, especially in the head and neck skin. Clinical manifestations of cSCC range from relatively low risk in situ SCC to high-risk malignancies with local or systemic spread [2]. Patients with cSCC have a 2–4% risk of metastatic disease to local lymph nodes. Nevertheless, the risk varies according to the tumor typeas well asits physical location and concurrent medical conditions [3]. Research has focused on upgrading risk stratification for patients without metastatic diseases, which has led to adjustments invalidation and staging guidelines [4]. The head and neck area is particularly vulnerable to sunlight exposure and ultraviolet radiation, leading to early dissemination in the regional lymph nodes (ipsilateral submandibular, sublingual, and parotid) [5]. cSCC typically metastasizes locally within 1–2 years post detection. Immunosuppression is another significant risk factor for cSCC [6]. Recipients of solid organ transplants are up to 200 times more likely to develop cSCC than other individuals. Also, patients with chronic lymphoblastic leukemia or HIV infection have an increased risk of cSCC [7]. This study examines the various factors that influence cancer patients’ survival, with a focus on the impact of immunosuppression. It effectively identifies key contributors to immunosuppression, with second cancer, diabetes, lymphoma, HIV, lupus, scleroderma, psoriasis, and leukemia being the causes studied in the cohort. The relationship between immune status and clinical outcomes is well established, implying that immunosuppression exacerbates negative prognostic factors such as age and tumor stage.

Most individuals with cSCC have a localized and relatively safe disease that can be treated with surgical resection, such as Mohs micrographic surgery. The micro excision or wide excision allows for disease-free rates of almost 90% after 5 years [8]. While the average rate of mortality for patients with cSCC is roughly 1–3%, the total number of deaths is predicted to be very similar to that of melanoma [9]. Advanced cSCC patients have a poor outcome due to significant recurrence rates [10] (nearly 50% with extensive perineural invasion) [11], metastasis rates (32.8% with low-grade differentiated tumors), and mortality rates (a 5-year and 10-year survival rate of 60% and <20%, respectively, in patients with locally lymph node involvement, and an average 10-year survival rate of less than 10 percent in those with distant metastases), even with adjuvant radiotherapy or chemotherapy [12]. This study aimed to answer clinical concerns regarding cSCC treatment, including detecting patients who are not suitable for surgery, providing an outline for current treatment options, and developing reliable recommendations for cSCC tumor evaluation, treatment, and follow-up. A multidisciplinary team plays an important role in managing advanced cases [13].

## 2. Materials and Methods

**Figure 1:**
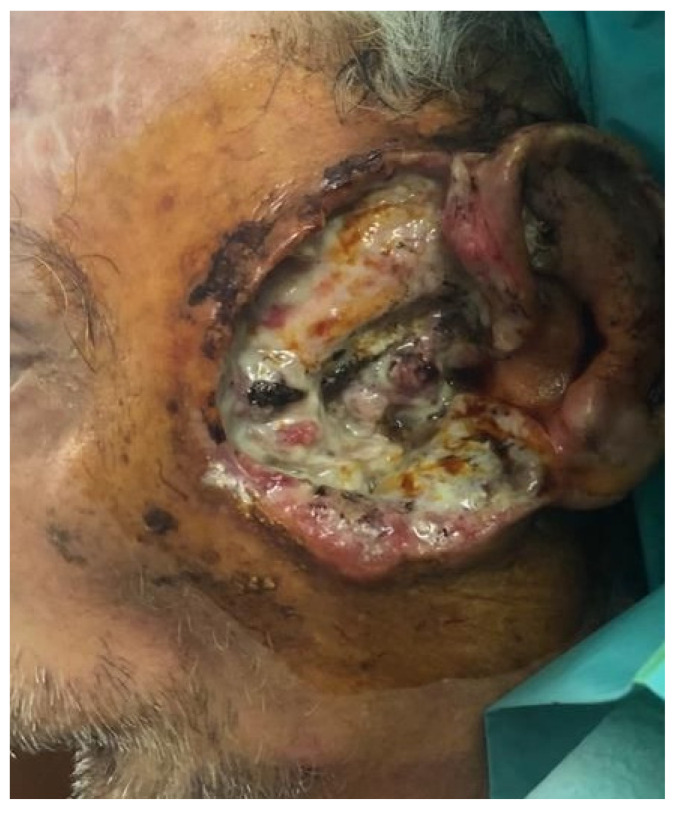
Locally advanced stage 3 preauricular squamous cell carcinoma with bone invasion of a immunosuppressed patient (type 2 diabetes treated with insulin for 15 years).

**Figure 2:**
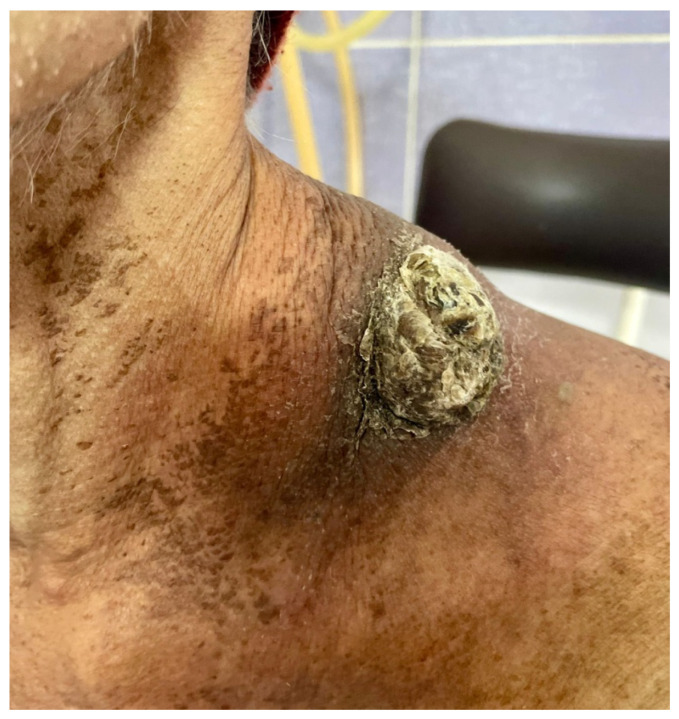
Advanced stage 4 cSCC of the neck as a second malignancy after non-small lung carcinoma (adenocarcinoma).

The relationship between immunosuppression and disease-specific outcomes in individuals with cutaneous squamous cell carcinoma is evaluated in this cohort research. Immunocompromised patients have a greater tendency to develop cSCC [14] and frequently have severe, multifocal illness [15]. Compared to patients without immunosuppression, those with immunodeficiency are more likely to experience lymphovascular invasion, extracapsular extension, and poorly differentiated disease [16]. These patients are also more likely to have larger primary tumors, achieve nodal disease, and have early cutaneous invasion (Figure 1 and Figure 2). The purpose of this research is to examine the outcomes of patients with and without immunosuppression following treatment for cHNSCC [17].

### 2.1. Methods

A literature review [18,19,20,21,22,23] was performed to find English-language publications in the electronic databases PubMed and ScienceDirect. We found numerous clinical trials and research regarding the outcomes of cHNSCC (Table 1).

We examined clinical cases of patients from Saint Apostle Andrew Emergency County Clinical Hospital in Galati, Romania, over a six-year period from 1 January 2018 to 1 April 2024. Patients with a pathologically confirmed diagnosis of cHNSCC who were at least 18 years old were eligible to participate in the trial. The recruitment period was of 16 months, between 1 January 2018 and 30 April 2019. Exclusion criteria were patients under 18 years old, prior radiotherapy to the region of the investigated cancer, patients who have previously been treated with chemotherapy for the evaluated tumor, recurrent cHNSCC, and patient refusal to participate in the study due to lack of trust in the trial process or personal beliefs (no patient refused to participate). We found 68 eligible cases. All patients signed consent forms that included agreement for using their personal medical information and/or pictures. Patients with other malignancies, solid organ transplants, stem cell transplants, systemic lupus erythematosus, rheumatoid arthritis, inflammatory bowel disease, scleroderma, psoriasis, lymphoma, leukemia, type 1 or type 2 diabetes, HIV or AIDS, or other hematoproliferative disorders were classified as being immunosuppressed. Clinical history, treatment history, and diagnostic data were obtained from HIPOCRATE patients’ personal health data used in the regional hospital. From all selected cases, we discovered 21 immunosuppressed patients (group A). Five of the patients were diagnosed on-site. Their immunosuppressed pathologies were noted in Table 2. It was considered that none of the other patients had immunosuppression (group B). None of the patients had received chemotherapy or radiotherapy for their cSCC, although one patient in the A group and eight patients in the B group had undergone surgery. The outcome measure was overall survival, defined as the time measured in months from initial medical examination to death due to any cause or loss to follow-up, as well as the three- and five-year survival rates. Clinical features of the patients are pointed out in Table 2.

### 2.2. Impact of Age on Survival

The study divides patients into two age categories: those under 45 and those beyond 45. Younger immunosuppressed patients (<45 years) have a higher median survival rate (19 months) compared to individuals over 45 (15 months). This conclusion is consistent with the frequent assumption that younger patients typically have better physiological reserves, allowing them to more easily endure illness progression and accompanying treatments. Immunocompetent patients display a similar pattern, but with more significant median survival disparities (37 months for <45 years and 31 months for >45 years). This suggests that immunological competence may provide an additional survival benefit, particularly among younger people [24].

### 2.3. Sex-Based Differences

Sex differences in survival outcomes are also notable. Immunosuppressed males have a significantly lower median survival grade (14 months) compared to females (17 months), suggesting that female patients in this group might possess inherent advantages, possibly due to hormonal or genetic differences that influence immune response and disease progression. In the immunocompetent cohort, females outlive males (41 months vs. 22 months). This supports the concept that sex-based biological variations may lead to better outcomes, particularly in loss of immune function [25].

### 2.4. Tumor Stage and Survival

Staging, as expected, is critical for survival. Patients with early-stage tumors (TNM stages 1–2) have much better outcomes than those with advanced stages (TNM stages 3, 4). Immunosuppressed people with early-stage disease have a 21-month survival rate, which drops dramatically to 11 months for advanced stages. This sharp drop highlights the combined detrimental impact of advanced tumor stage and immunosuppression on prognosis. Similarly, immunocompetent patients with early-stage disease have much longer survival (43 months) than those with advanced-stage disease (18 months). This trend emphasizes the significance of early detection and intervention, which is especially crucial in immunocompromised individuals [26].

### 2.5. History of Tumor Surgical Treatment

The survival advantage provided by surgical intervention is clear. Immunosuppressed patients with a history of tumor surgical treatment had a 16-month survival rate compared to the cohort average, but immunocompetent patients with a similar surgical history have the longest survival rate, of 51 months. This finding suggests that, when possible, surgical intervention has a significant impact on long-term outcomes and is especially beneficial to immunocompetent people [27].

### 2.6. Immunosuppression as a Key Determinant

Throughout all clinical characteristics, immunosuppressed patients have consistently lower survival rates than their immunocompetent peers. For example, the overall survival time for immunosuppressed individuals ranges from 11 to 21 months depending on the characteristic, whereas that for immunocompetent individuals ranges from 18 to 51 months. This difference emphasizes the critical role of immune competence in determining survival in patients with advanced illnesses [28].

## 3. Results

**Figure 3:**
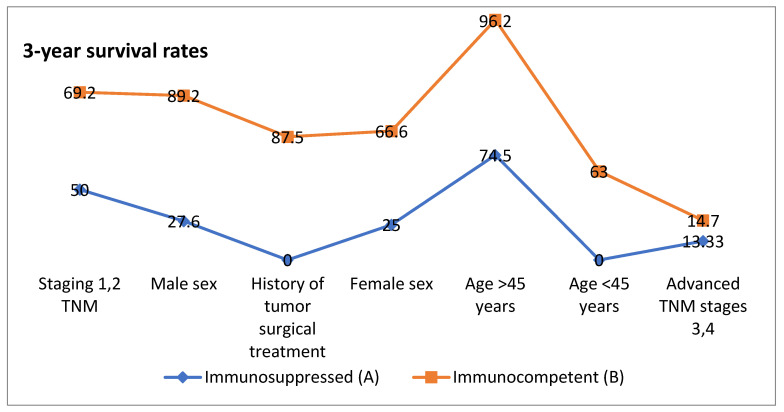
Differences in 3-year survival rates between immunocompetent and immunosuppressed groups based on clinical features.

**Figure 4:**
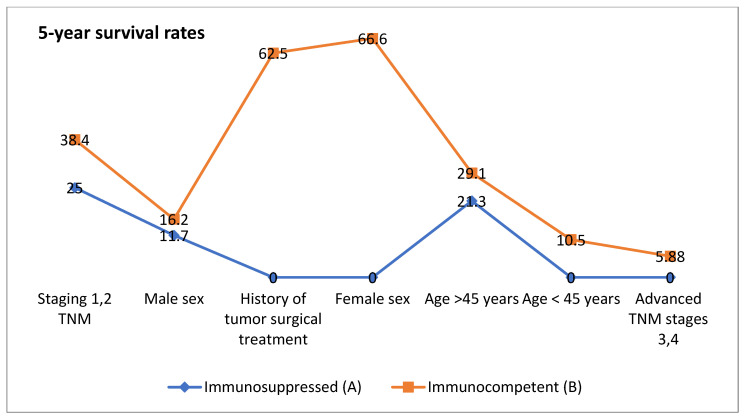
Differences in 5-year survival rates between immunocompetent and immunosuppressed groups based on clinical features.

This study underscores the multifactorial determinants of survival, including age, sex, tumor stage, surgical intervention, and immune status (Figure 3 and Figure 4). In this cohort, the most common cause of immunosuppression was other types of cancer (*n* = 7 [30.4%]), followed by type 1 or type 2 diabetes (*n* = 6 [26%]) and lymphoma (*n* = 3 [13%]). Other causes of immunosuppression included leukemia, psoriasis, scleroderma, lupus, and HIV. Two patients had more than one cause of immunosuppression. The condition emerges as a significant adverse factor, amplifying the impact of other negative prognostic indicators such as advanced age and higher tumor stages. These results highlight the importance of tailored treatment strategies that take into account both immune status and clinical characteristics in order to achieve successful outcomes. It is critical to consider the *p*-values separately but also with the clinical relevance. Statistically significant *p*-values (typically less than 0.05) indicate that the differences in median survival, overall survival rates, and other outcomes between Group A and Group B are significant, although a statistically significant outcome does not always imply a clinically significant difference.

The majority of patients received primary radiotherapy at Saint Andrew Hospital of Galati (A group *n* = 18 [85.7%], B group no = 33 [70.2%]; difference −15.4%), followed by second post-surgical therapy (A group *n* = 1 [4.7%], B group *n* = 8 [17.02%]; difference 12.32). Although the current guides accept immunosuppression as a high-risk feature of cSCC, the most recent staging systems, for example, the eighth edition of the American Joint Committee on Cancer and the Brigham and Women’s Hospital, do not include immunosuppression status as a risk factor [29]. Further research into immune-modulating therapies and early-detection strategies could lead to additional benefits, particularly for high-risk populations.

## 4. Discussion

The graphs compare 3-year and 5-year survival rates between immunosuppressed and immunocompetent individuals based on various factors. Overall, immunocompetent individuals have higher survival rates across most categories, although immunosuppressed individuals under 45 years of age have a higher survival rate than those over 45 years of age.

Radiological imaging, including CT, MRI, PET, and ultrasound, is utilized to detect subclinical nodal spread [30]. CT can detect central nodal tissue necrosis, skull base invasion, cartilage involvement, and extracapsular spread, which is useful [31]. MRI is crucial for detecting neuronal tumors, determining tissue direction, and differentiating tumor tissue from muscle. Imaging instruments can help plan the treatment for cancers that have spread to deeper tissues, including, locally, lymph nodes, the parotid gland, bone, neurological system [32]. Clinicians experience challenges predicting the probability of metastasis and mortality for cHNSCC due to the lack of solid prognostic criteria. A lack of verified research has prevented the development of therapeutic standards [33]. Over the course of five years following treatment, when 95% of recurrences and metastases are identified, routine follow-ups should occur every three to six months. During these visits, a thorough examination of the skin, entire body, and lymph node region should be conducted [34].

Our findings support the initiative of including immunosuppression as an important prognostic factor. The discussion on the importance of tailored treatment strategies addresses a significant gap in current clinical guidelines, specifically, the omission of immunosuppression status in the latest staging systems. Further research into immune-modulating therapies, underscoring the need for including immunosuppression as an important prognostic factor to improve outcomes for high-risk populations, is required.

### Staging Systems and Risk Stratification in cSCC

Two verified staging systems for cSCC are accessible. Clinicians frequently apply either the American Joint Committee on Cancer or the Brigham and Women’s Hospital system for staging [35]. Current clinical guidelines have no formal criteria, yet some recommendations are used in order to assist tumor staging [36]. Evidence-based risk factors include tumor size (~2 cm) and thickness, tissue invasion, histological differentiation, the existence of desmoplasia, perineural, lymphovascular, muscular, or bone invasion, and lymphocyte infiltration, which can be hard to determine and evaluate [37]. Sentinel lymph node samples have been studied as a prognostic factor; however, data from relatively small trials are limited. The technique’s predictive significance has yet to be determined [38].

Furthermore, patient characteristics associated with bad outcomes in cSCC include immunosuppression, tumor location (lip, ear, temple, vermillion, periorbital, and anogenital), associated diseases (albinism, xeroderma pigmentosum, and epidermolysis bullosa), the involvement of a high number of lymph nodes, and/or the extracapsular size of tumor in lymph nodes [39].

## 5. Management of cSCC

### 5.1. Radiotherapy

Radiotherapy has high response rates, particularly for small tumor size and external skin damage in immunocompetent subjects. Furthermore, the functional and esthetic outcomes of radiotherapy are typically outstanding [40]. For patients with cSCC who do not qualify for surgical treatment, guidelines recommend primary radiation therapy. Adjuvant radiation therapy following surgery may be an alternative for cSCC tumors with uncertain surgical margins, important nerve involvement, perineural invasion, a high risk of regional or distant metastases, or multiple lesions [41]. Combining surgery and radiation therapy starting from 60 Gy enhanced local control for patients with parotid metastatic cancer by 86%, compared to 47% with radiation therapy alone [42]. Radiotherapy causes inflammation in the tumor microenvironment due to DNA damage. Tumor cells upregulate the PD-L1 receptor through an elaborate route that includes DNA damage signaling, IFNγ signaling, and the EGFR pathway [43]. This method enhances PD-L1 expression according to radiation exposure, both in vitro and in vivo. Radiation-induced inflammation attracts T cells into the tumor microenvironment, leading to a rise in PD-L1 expression, indicating a tumor’s protective mechanism against cell death [44]. This could be a clinical strategy for improving the immunotherapy response. Another possible consequence of high-dose radiation is the abscopal effect, which is the alteration of the body’s immune response that results in a decrease in other metastatic locations outside of the radiation area. In rare instances, this impact may increase survival [45]. The radiation effect on the molecular mechanism is complicated, although it is believed to be mediated by CD8 + T cells. According to a case study, a patient with multiple synchronous cSCC experienced spontaneous regression of the non-irradiated lesions after receiving brachytherapy for one of them. As a result, the synergy of immunotherapy and radiation may result in a better response [46]. However, there is still room for improvement in the treatment of high-risk patients through surgery and adjuvant radiotherapy [47].

### 5.2. Immunotherapy

Treatment choices and prognosis are heavily influenced by tumor characteristics and patient factors [48]. Before immunotherapy emerged, the only systemic treatment options for HNSCC were cytotoxic platinum-based chemotherapy and targeted therapies that targeted the epidermal growth factor receptor (EGFR) [49]. Immunotherapy has transformed the management of advanced and metastatic cSCC with its 50% response rate, excellent tolerability, and durable disease control [50]. In 2018, the United States Food and Drug Administration (FDA) granted approval for Cemiplimab as the first immunotherapy agent to treat metastatic or locally advanced cutaneous squamous cell carcinoma (CSCC) that has not responded to curative therapies [51]. The immune system’s response to cancer cells is largely influenced by immune checkpoint proteins. T-cell activation occurs in two stages: first, the recognition of peptides by the T-cell receptor, and second, the interaction of partner proteins on tumor cells with co-regulatory proteins (immune checkpoints) displayed on T cells [52]. When these immune checkpoints are activated, they can have either a stimulating or inhibiting effect on the immune system [53]. Typically, immune checkpoints help to ensure a balanced immune response while safeguarding healthy tissues and preventing excessive immune activation, which can lead to autoimmune diseases. Cemiplimab is a monoclonal antibody that specifically targets PD-1 [54]. It has demonstrated an acceptable safety profile with a discontinuation rate of only 7% and adverse event rates similar to those of other anti-PD-1 therapies [55]. Another immunotherapy option is pembrolizumab, which is approved for patients with locally advanced, metastatic, or recurrent head and neck squamous cell carcinoma (HNSCC) who are not suitable for radiation or surgical intervention [56]. Pembrolizumab is generally well tolerated and shows favorable outcomes. Immune-related adverse effects, particularly cutaneous reactions like the rare Stevens-Johnson syndrome and toxic epidermal necrolysis, have typically been treated with glucocorticoids [57].

### 5.3. Anti-EGFR (Epidermal Growth Factor Receptor)

Anti-EGFR treatment plays a significant role in the management of advanced HNSCC [58]. EGFR is a cell surface receptor that, when activated, promotes cell proliferation, division, and survival [59]. Many head and neck cancers over-express EGFR, resulting in uncontrolled cell proliferation. Anti-EGFR therapies, such as cetuximab, work by binding to the EGFR on cancer cells, blocking its activation, and inhibiting downstream signaling pathways that promote tumor growth [60]. Anti-EGFR treatments are typically used in combination with chemotherapy and radiation therapy for patients with locally advanced HNSCC, or as a stand-alone treatment for those with recurrent or metastatic cases who are not candidates for chemotherapy [61]. Anti-EGFR therapies have been shown to improve both overall survival and progression-free survival in patients with advanced HNSCC. They may also improve the effects of radiation therapy, resulting in more effective treatment outcomes [62].

### 5.4. Complications

Nearly 2500 cases of aggressive tumors are discovered each year, with significant rates of recurrence, increased morbidity, metastasis, and death. The insufficient control of regional metastases contributes to the high rate of recurrence and regional metastasis during the first course of treatment, as well as the lack of detecting such aggressive lesions [63]. The eyes, ears, or nose function may be affected by surgical treatment techniques for aggressive cHNSCC, which could have visible cosmetic and social impacts. A significant amount of facial repair is necessary in these situations [64]. The quality of life eventually becomes affected by such aggressive carcinomas, leading to additional complications [65]. By creating e-health applications, such as the self-monitoring of the health-related quality of life (HRQOL)and self-help therapies, significant efforts are being made to enhance the HRQOL for patients with HNSCC [66]. These developments have the potential to empower HNSCC survivors and provide supportive care in a sustainable manner [67]. Other data sources, such as biomarkers and objective measurements (gathered, for instance, via devices that are wearable, like a smartwatch, that records and analyzes activity), can improve the measurement accuracy of the HRQOL by patient-reported outcome measures. Additionally, new data-driven analytics can be applied in order to study and improve HRQOL expectations. Providing recruited HRQOL data that are effectively accessible to patients and doctors is one of the current main challenges as well [68]. This will help with decision-making post-therapy and identifying the benefits, particularly for high-risk populations.

## 6. Limitations of This Study and Future Perspectives

The accuracy of documentation is one of the study’s limitations, and important data could possibly not be available or accessible since all were obtained from only one county hospital medical archive. Due to the varied nature of the causes of immunosuppression in this group (Table 3), we were unable to precisely determine and compare the severity or duration of immunosuppression. Furthermore, this study was conducted at a regional general hospital, which may limit the external validity of the findings. Subgroup analysis was completed by including a variety of immunosuppressive causes, which improved our understanding of how various forms of immunosuppression affect overall survival. The present study highlights the need for further research in patients with cHNSCC and compromised immunity. In the subgroup analysis, the patient with HIV had the poorest prognosis in the B group. Although surgery remains the primary treatment for these patients, these findings indicate that a better understanding of adjuvant treatment options in these high-risk patients is required to improve their disease outcomes.

### Future Perspectives

Immune checkpoint inhibitors (ICIs) selecting PD-1 and PD-L1 have improved cSCC treatment, particularly for patients with locally advanced and metastatic disease. Current trials primarily use ICIs, including three new agents designed to suppress PD-1 and PD-L1 [69]. New research is looking into the efficiency of neoadjuvant, adjuvant, or mixed (neoadjuvant and adjuvant) ICI therapy alongside surgery and/or RT [70]. Some additional treatment agents are being used with prior ICI therapy, including drugs that activate IL-2, IL-7, IL-15, as well asTLR-7/8 and TLR-9, or inhibit C5a and EGFR [71]. These agents provide individuals suffering from advanced disease with lower therapeutic toxicity, a longer-lasting response after discontinuation [72], and higher survival rates than traditional chemotherapy agents (cisplatin, carboplatin, fluorouracil, bleomycin, doxorubicin, and methotrexate) [73]. The ICI treatment is shorted by the rejection of the transplanted organ, which can occur in up to42% of renal transplants [74]. As the prevalence of cSCC rises and more patients have high-risk factors, clinicians must also evaluate the potential of immunosuppression and include up-to-date treatment options.

## Figures and Tables

**Table 1:** Trials evaluating different outcomes for cHNSCC.

Trial Registration Number	Clinical Condition	Number of Participants	Time Period	Results
NCT01979211 [18]	Locally advanced HNcSCC	24	2013–2022	67.5% 5-year median overall survival
NCT02643303 [19]	Advanced, measurable, biopsy-accessible head and neck cancer	58	2015–2022	338 days median overall survival
NCT03229278 [20]	Solid malignancy or lymphoma that is metastatic or unresectable	14	2017–2022	21% 6-month progression-free survival rate
NCT04502888 [21]	HNcSCC	18	2020–2022	Not submitted
NCT00193895 [22]	HNcSCC	321	2005–2016	83% Freedom from locoregional relapse
NCT04966481 [23]	HPV-unrelated recurrent or metastatic head and neck squamouscell carcinoma	81	2022–2027	9.7 months median overall survival

**Table 2:** Survival rates and overall survival based on immunosuppression and different clinical aspects. Group A: immunosuppressed patients; Group B: immunocompetent patients.

CLINICAL Characteristics	Group A	Group B	Median Overall Survival (Months)		3-Year Survival Rates% (Months)		5-Year Survival Rates % (Months)	
			A	B	A	B	A	B
Age < 45 years	2	10	19	37	0	63	0	10.5
Age > 45 years	19	37	15	31	74.5	96.2	21.3	29.1
Male sex	17	41	14	22	27.6	89.2	11.7	16.2
Female sex	4	6	17	41	25	66.6	0	66.6
Staging 1,2 TNM	6	13	21	43	50	69.2	25	38.4
Advanced TNM stages 3,4	15	34	11	18	13.33	14.7	0	5.88
History of tumor surgical treatment	1	8	16	51	0	87.5	0	62.5
*p* Value			*p* = 0.005		*p* = 0.013		*p* = 0.040	

**Table 3:** Immunosuppressive medical conditions evaluated in the study.

Immunosuppression Diseases	Number of Cases
Other malignancies	7
Systemic lupus erythematosus	1
Type 1 or type 2 diabetes	6
HIV	1
Scleroderma	2
Psoriasis	2
Lymphoma	3
Leukemia	1

## Data Availability

The raw data supporting the conclusions of this article will be made available by the corresponding authors on request.
